# German Conference on Chemoinformatics 2010 – organizers' notes

**DOI:** 10.1186/1758-2946-3-S1-I1

**Published:** 2011-04-19

**Authors:** Frank Oellien, Uli Fechner, Thomas Engel

**Affiliations:** 1 GDCh-CIC Division Chair, Intervet Innovation GmbH, Zur Propstei, 55270 Schwabenheim, Germany; 2 GDCh-CIC Division Board Member, Beilstein-Institut zur Förderung der Chemischen Wissenschaften, Trakehner Str. 7-9, 60487 Frankfurt, Germany; 3 GDCh-CIC Division Co-Chair and Conference Chair, Fakultät für Chemie und Pharmazie, Universität München, Butenandtstr. 5-13, 81377 München, Germany

## 

The Chemistry-Information-Computers (CIC) division [1] of the German Chemical Society (GDCh) invited the chemoinformatics and modeling community from the 7^th^ to the 9^th^ November 2010 to Goslar, Germany, to participate in the **6th German Conference on Chemoinformatics** (GCC 2010). The international symposium addressed a broad range of modern research topics in the realm of computers and chemistry. The conference focused on recent developments and trends in the fields of

• *Chemoinformatics and Drug Discovery*

• *Chemical Information*, *Patents and Databases*

• *Molecular Modeling*

• *Computational Material Science and Nanotechnology*

In addition, contributions from other research areas of Computational Chemistry were welcome.

**Figure 1:**
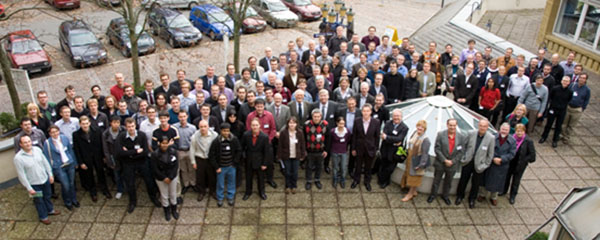
Participants of the 6. German Conference on Chemoinformatics, November 7–9, 2010, Goslar, Germany.

**Figure 2:**
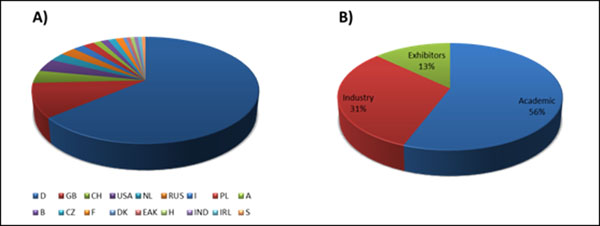
A) 156 participants from 18 nations attended the GCC2010. Most participants (99) were from Germany followed by 16 attendees from the United Kingdom. Participants from the USA and Switzerland followed on the third position with 6 attendees each. B) 56% of the participants work at academic institutes, 31% in industry and 13% are exhibitors.

Similar to other conferences in the Life Science sector the German Conference on Chemoinformatics was faced with a decreasing number of participants in 2010. This is assumed to be mainly caused by travel cost cuts in industry due to the global economic crisis. Nevertheless, a large number of scientists (156 participants, Figure 1) from 18 nations were attracted by the excellent scientific program established by the scientific advisory board and attended the GCC 2010. The large number of attendees from countries other than Germany demonstrates that the conference is an internationally well-established event in the global Chemoinformatics and Modelling community (see Figure 2A). Despite the travel situation in industry, a good ratio between academic institutes, industry and exhibitors could be achieved (see Figure 2B).

Traditionally, the conference was opened by a *“Free-Software-Session”* on Sunday afternoon right before the official conference opening at 5 pm. Six Open Source projects were presented in *“Free-Software-Session”*. This was the largest number of contributions to this session in the history of the GCC. The audience followed presentations of PostgreSQL (Bayer Business Services), OCHEM (Helmholtz Zentrum München), Knime (University of Konstanz), Indigo & Ketcher (SciTouch), and MosGrid (MosGrid Consortium). The *“Chemoinformatics Market Place”* took place at the same time and included software tutorials by the Chemical Computing Group (*“New Features of MOE 2010”*) and BioSolveIT (*“Fragment-Based Lead Design in Teams”*).

The scientific program started with an entertaining and enthralling evening lecture giving by Colleen Fitzpatrick (*“Hand in Snow”*). In addition, the program of the following two days included plenary lectures of six well known invited speakers from industry and academia (Wolfgang Guba, Roche, Swiss; Andrew R. Leach, GSK, UK; Hans Fraaije, Leiden University, The Netherlands; Jürgen Gmehling, Oldenburg University, Germany; Gerhard Klebe, Marburg University, Germany; Holger Gohlke, Düsseldorf University, Germany), 17 lectures as well as 43 poster presentations.

**Figure 3:**
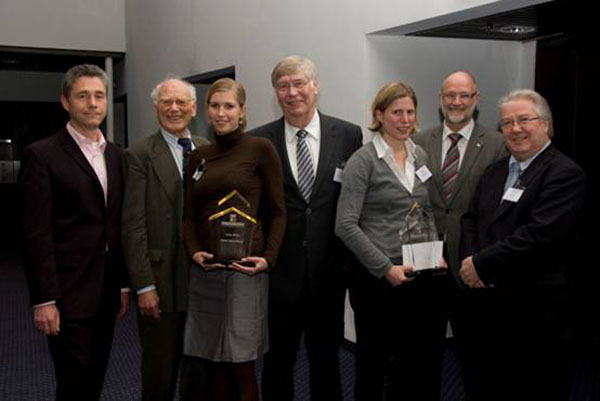
FIZ CHEMIE Berlin Awards 2010 and GDCh Gmelin-Beilstein-Denkmünze Award: from left to right, Frank Oellien (Chair of the GDCh-CIC division), Ulfert Onken (Laudatory Gmelin-Beilstein-Denkmünze Award), Karen Schomburg (FIZ CHEMIE Berlin Awardee, best master thesis), Jürgen Gmehling (Gmelin-Beilstein-Denkmünze Awardee), Simone Fulle (FIZ CHEMIE Berlin Awardee, best PhD thesis), Michael Dröscher (President of the German Chemical Society), Rene de Planque (Head of the FIZ CHEMIE Berlin).

Besides the scientific program a special highlight of the conference were the awards (see Figure 3). In contrast to the last GCC in 2009, two different awards could be handed over for excellent scientific work this year. Prof. Jürgen Gmehling from the University of Oldenburg was presented with the Gmelin-Beilstein-Denkmünze [2] award for his groundbreaking contributions in the field of compound databases and fragment methods for the prediction of chemical properties used in technical chemistry. The laudatory speech was given by Prof. Onken and the award was handed over by the president of the German Chemical Society Prof. Michael Dröscher. Furthermore, the FIZ-CHEMIE-Berlin awards [3] took place on Monday afternoon. The CIC division awards this price each year to the best diploma thesis and the best PhD thesis in the field of Computational Chemistry. The price for the PhD thesis was awarded to Dr. Simone Fulle from the group of Prof. Dr. Holger Gohlke, Goethe University of Frankfurt for her dissertation “*Constraint counting on RNA and ribosomal structures: Linking flexibility and function*”. The award for the best diploma thesis was given to Karen Schomburg from the group of Prof. Matthias Rarey, University of Hamburg for her excellent master thesis with the title “*Visualization of molecular subgraph pattern using the example of SMARTS pattern*”.
